# Supplementary material for: Spatial transcriptomics reveals molecular dysfunction associated with Lewy pathology
Source: bioRxiv. 2023 May 17:2023.05.17.541144. Preprint. [Version 1] doi: 10.1101/2023.05.17.541144 (PMC10245657; doi:10.1101/2023.05.17.541144)
Supplement: Supplement 1 [file NIHPP2023.05.17.541144v1-supplement-1.pdf]

bioRxiv preprint doi: https://doi.org/10.1101/2023.05.17.541144; this version posted May 17, 2023. The copyright holder for this preprint (which was not certified by peer review) is the author/funder, who has granted bioRxiv a license to display the preprint in perpetuity. It is made available under aCC-BY 4.0 International license.

Supplementary materials for

Spatial transcriptomics reveals cellular dysfunction associated with Lewy pathology

Thomas Goralski[1,2], Lindsay Meyerdirk[1,2], Libby Breton[1,2], Laura Brasseur[1], Kevin Kurgat[1,2], Daniella DeWeerd[1,2], Lisa Turner[3], Katelyn Becker[3], Marie Adams[3], Daniel Newhouse[4], Michael X. Henderson[1,2]*

[1]Department of Neurodegenerative Science, Van Andel Institute, Grand Rapids, MI 49503
[2]Aligning Science Across Parkinson's (ASAP) Collaborative Research Network, Chevy Chase, MD
[3]Van Andel Institute, Grand Rapids, MI 49503
[4]NanoString Technologies, Seattle, WA, USA

*Correspondence:
Michael X. Henderson
333 Bostwick Ave NE, Grand Rapids, MI 49503
(616) 234-5489
michael.henderson@vai.org

bioRxiv preprint doi: https://doi.org/10.1101/2023.05.17.541144; this version posted May 17, 2023. The copyright holder for this preprint (which was not certified by peer review) is the author/funder, who has granted bioRxiv a license to display the preprint in perpetuity. It is made available under aCC-BY 4.0 International license.

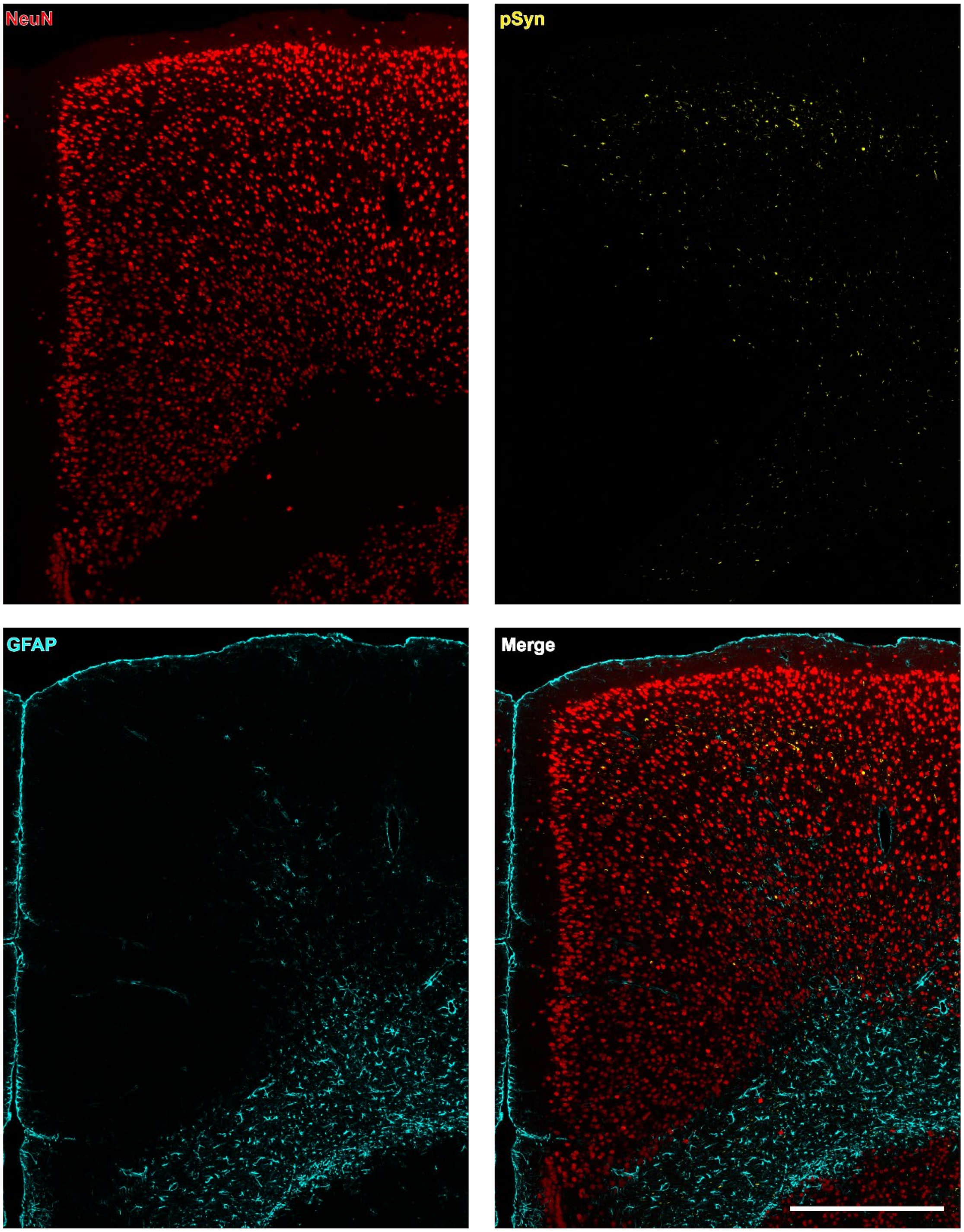

Supplementary Fig. 1. Representative cortex from α-synuclein PFF-injected mouse Three different proteins were stained for segmentation on the GeoMx DSP instrument. NeuN stains neuron cell bodies. pSyn stains α-synuclein pathology. GFAP stains for astrocytes. pSyn pathology is primarily observed in upper layer 5 and in layer 6 of cortex. Scale bar = 0.5 mm.

bioRxiv preprint doi: https://doi.org/10.1101/2023.05.17.541144; this version posted May 17, 2023. The copyright holder for this preprint (which was not certified by peer review) is the author/funder, who has granted bioRxiv a license to display the preprint in perpetuity. It is made available under aCC-BY 4.0 International license.

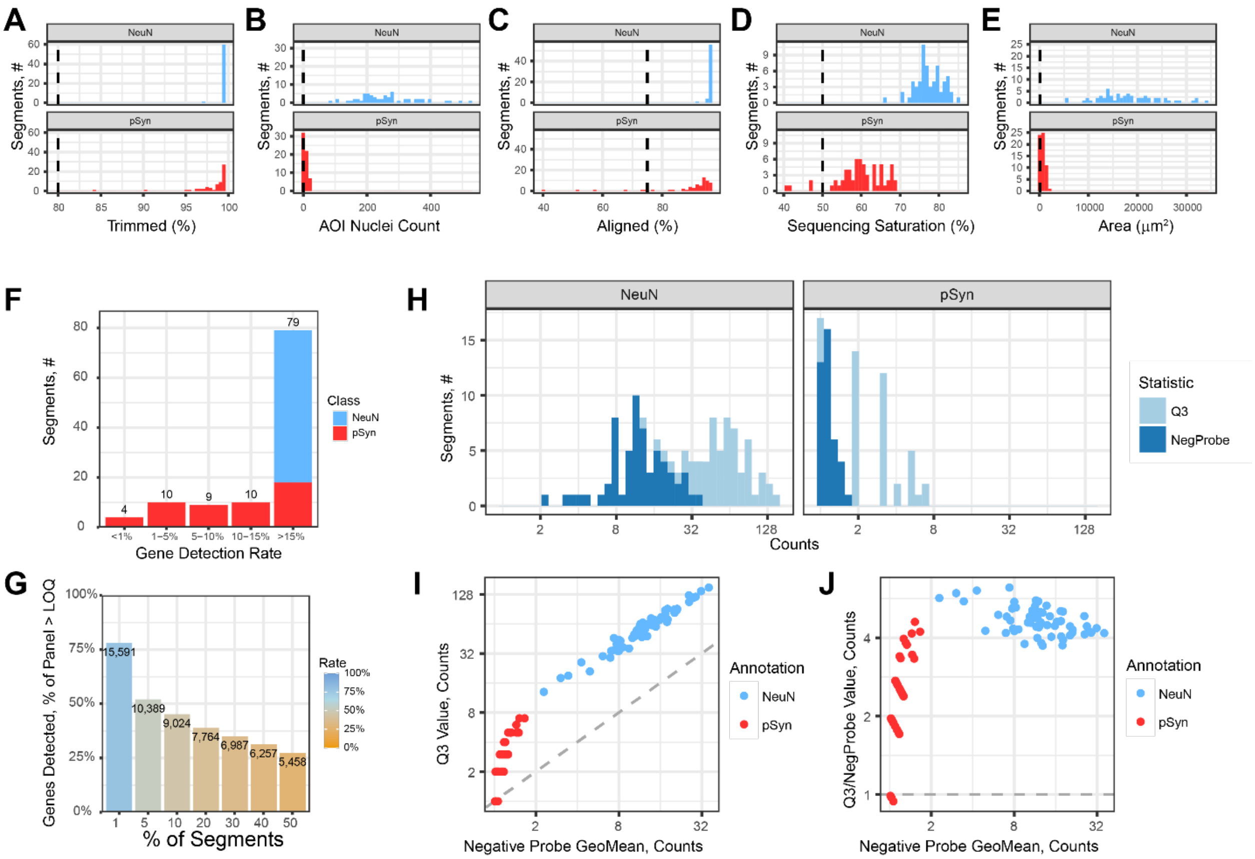

Supplementary Fig. 2 Quality control of mouse GeoMx data (A-E) Assessment of segments for quality based on spatial information and standard transcriptomics quality control metrics. **(F)** Number of segments with the given percentage of genes detected above the limit of quantification (LOQ). **(H)** Assessment of negative probe count values and Q3 values for each segment. **(G)** Assessment of the number of genes detected above LOQ in the given percentage of segments. **(I)** Comparison of the segments Q3 count value to the geometric mean of the negative probes. **(J)** Comparison of the segments Q3 values divided by their negative probes geometric mean to the negative probe geometric mean.

bioRxiv preprint doi: https://doi.org/10.1101/2023.05.17.541144; this version posted May 17, 2023. The copyright holder for this preprint (which was not certified by peer review) is the author/funder, who has granted bioRxiv a license to display the preprint in perpetuity. It is made available under aCC-BY 4.0 International license.

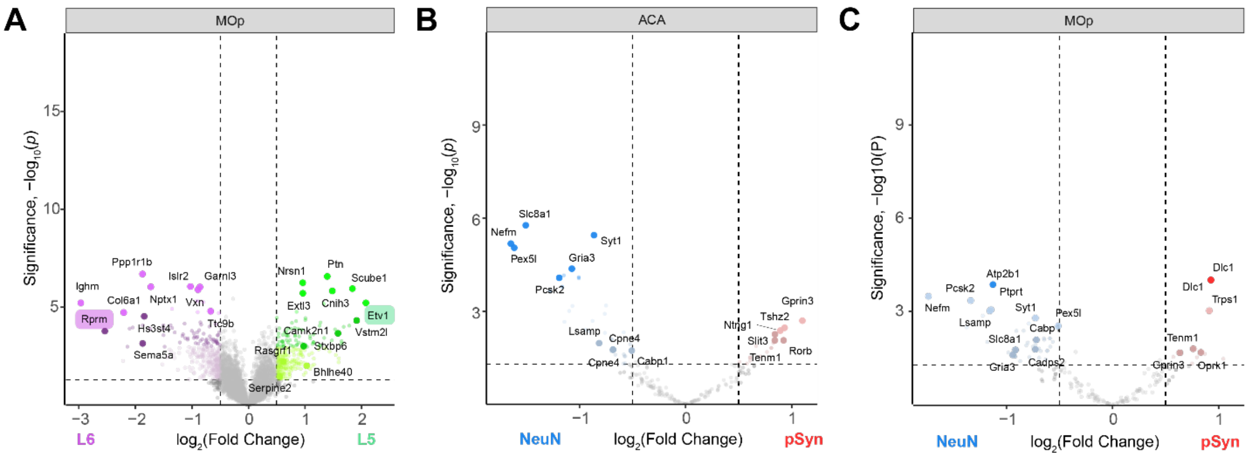

Supplementary Fig. 3. Differential gene expression by layer and cell types (**A**) Volcano plot comparing genes differentially expressed between NeuN segments in layer 5 and layer 6 of MOp identifies genes know to be differentially expressed in the different cortical layers. Two such genes are highlighted. (**B**) Volcano plot comparing genes differentially expressed between NeuN and pSyn segments in the ACA, but only comparing genes known to be differentially expressed by different cell types. (**C**) Volcano plot comparing genes differentially expressed between NeuN and pSyn segments in the MOp, but only comparing genes known to be differentially expressed by different cell types.

bioRxiv preprint doi: https://doi.org/10.1101/2023.05.17.541144; this version posted May 17, 2023. The copyright holder for this preprint (which was not certified by peer review) is the author/funder, who has granted bioRxiv a license to display the preprint in perpetuity. It is made available under aCC-BY 4.0 International license.

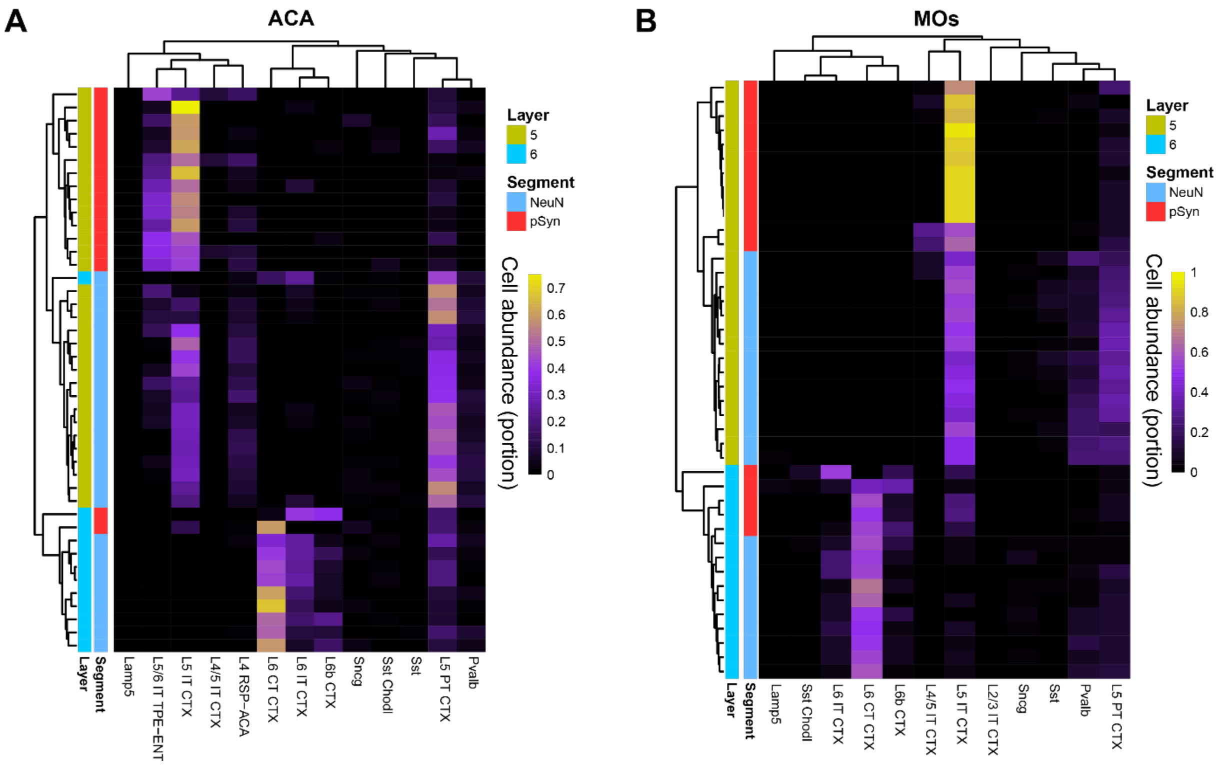

Supplementary Fig. 4. Cell deconvolution plots (**A**) The relative abundance of cell types was calculated via cell deconvolution for each segment in the ACA region. Layer 5 and layer 6 segments largely cluster separately, with NeuN and pSyn segments clustering within their respective layers. Layer 5 pSyn segments are largely L5 IT or L5/6 IT TPE-ENT cell types, while layer 5 NeuN segments show less abundance of L5 IT and a higher proportion of L5 PT neurons. Layer 6 pSyn segments are a mix of L6 CT and L6b neurons, while layer 6 NeuN segments are mostly L6 CT and L6 IT neurons. Similar results are seen for the MOs region (**B**).

bioRxiv preprint doi: https://doi.org/10.1101/2023.05.17.541144; this version posted May 17, 2023. The copyright holder for this preprint (which was not certified by peer review) is the author/funder, who has granted bioRxiv a license to display the preprint in perpetuity. It is made available under aCC-BY 4.0 International license.

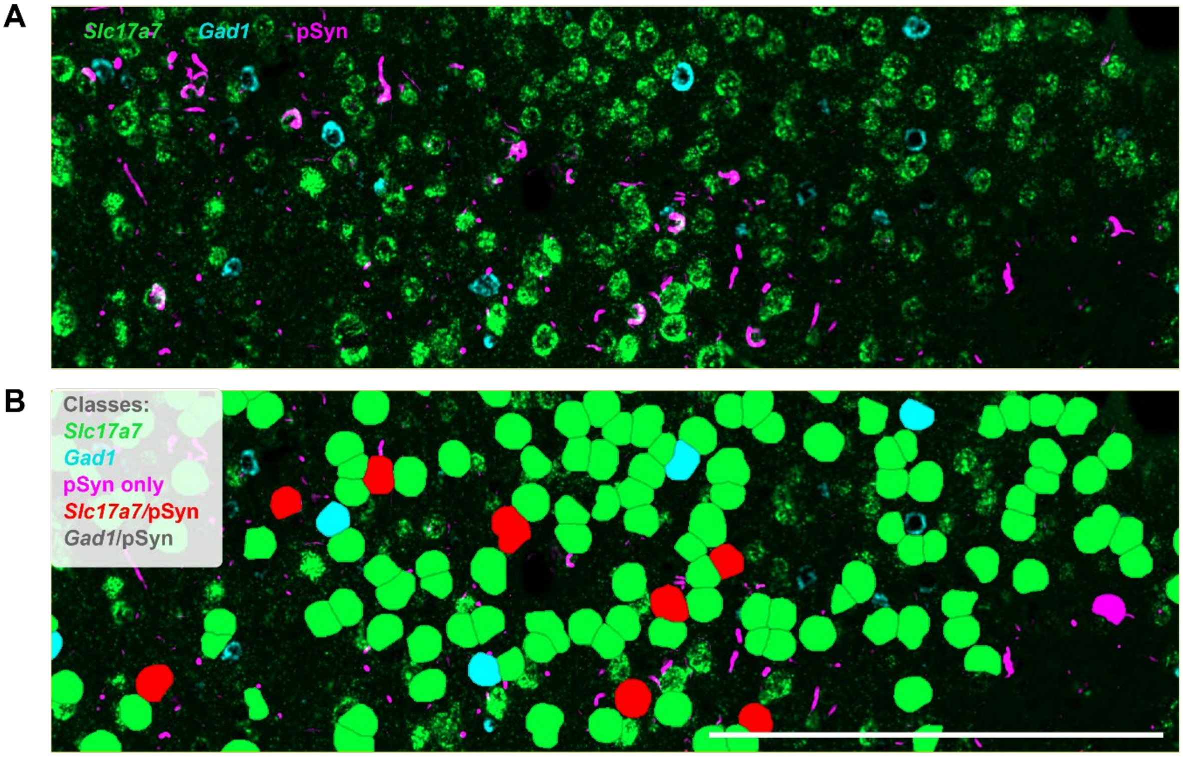

Supplementary Fig. 5 Cell classification in mouse cortex (**A**) Mouse cortex stained for RNAs *Slc17a7* and *Gad1* and protein pSyn. (**B**) The same image as in panel **A**, except cells were identified based on the presence of nuclear DAPI signal and classified based on the presence or absence of *Slc17a7*, *Gad1*, or pSyn in the cytoplasm. Cells with more than one marker were classified as having multiple markers. The overlay indicates the cell class. Scale bar = 250 µm.

bioRxiv preprint doi: https://doi.org/10.1101/2023.05.17.541144; this version posted May 17, 2023. The copyright holder for this preprint (which was not certified by peer review) is the author/funder, who has granted bioRxiv a license to display the preprint in perpetuity. It is made available under aCC-BY 4.0 International license.

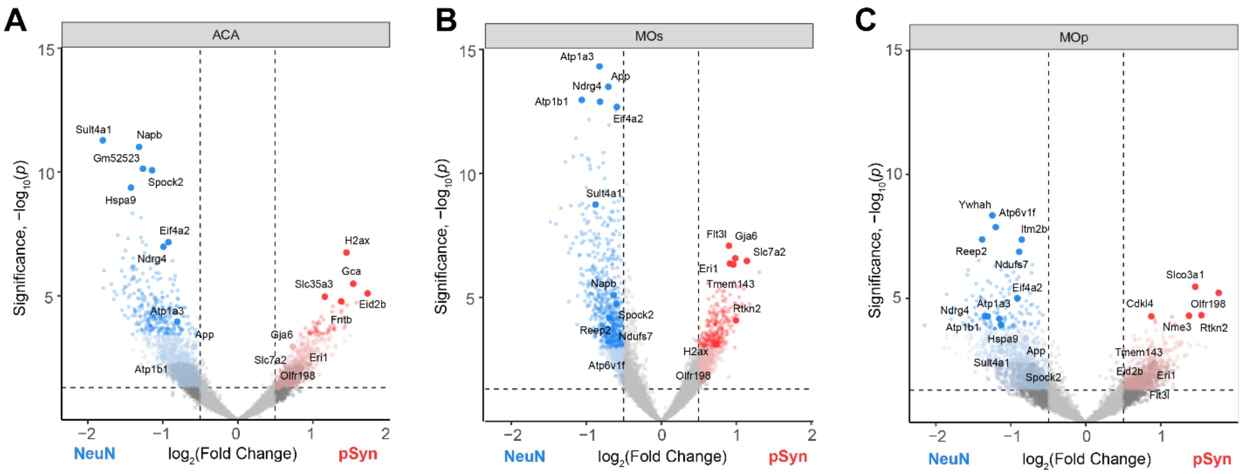

Supplementary Fig. 6. α-Synuclein inclusion-bearing neurons show conserved gene expression changes associated with cellular dysfunction in three cortical regions Volcano plot comparing genes differentially expressed between NeuN and pSyn segments in the ACA (**A**), MOs (**B**), or MOp (**C**) regions with the top 30 DEGs labeled.

bioRxiv preprint doi: https://doi.org/10.1101/2023.05.17.541144; this version posted May 17, 2023. The copyright holder for this preprint (which was not certified by peer review) is the author/funder, who has granted bioRxiv a license to display the preprint in perpetuity. It is made available under aCC-BY 4.0 International license.

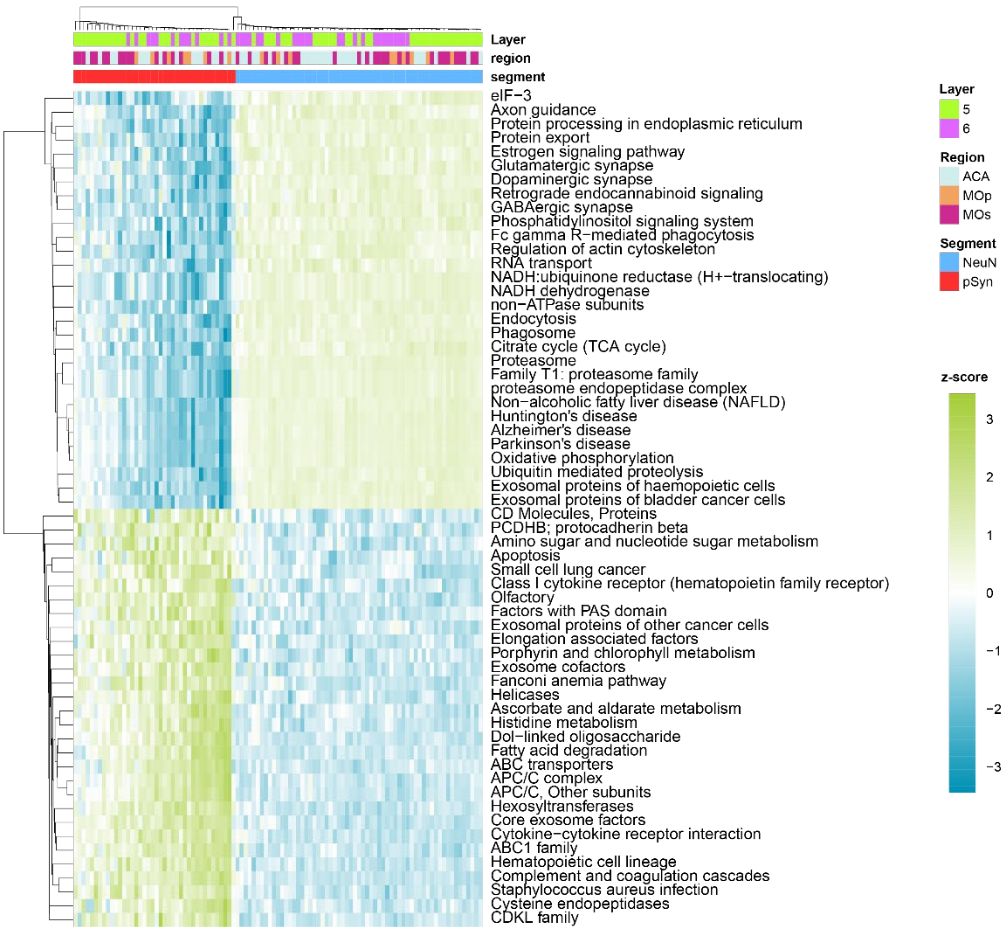

Supplementary Fig. 7. Mouse gene set enrichment analysis Gene set enrichment analysis was performed on NeuN and pSyn segments in from α-synuclein PFF-injected mice. Z-scores of individual segments are plotted for each pathway. The top 60 pathways enriched in either pSyn or NeuN segments are plotted.

bioRxiv preprint doi: https://doi.org/10.1101/2023.05.17.541144; this version posted May 17, 2023. The copyright holder for this preprint (which was not certified by peer review) is the author/funder, who has granted bioRxiv a license to display the preprint in perpetuity. It is made available under aCC-BY 4.0 International license.

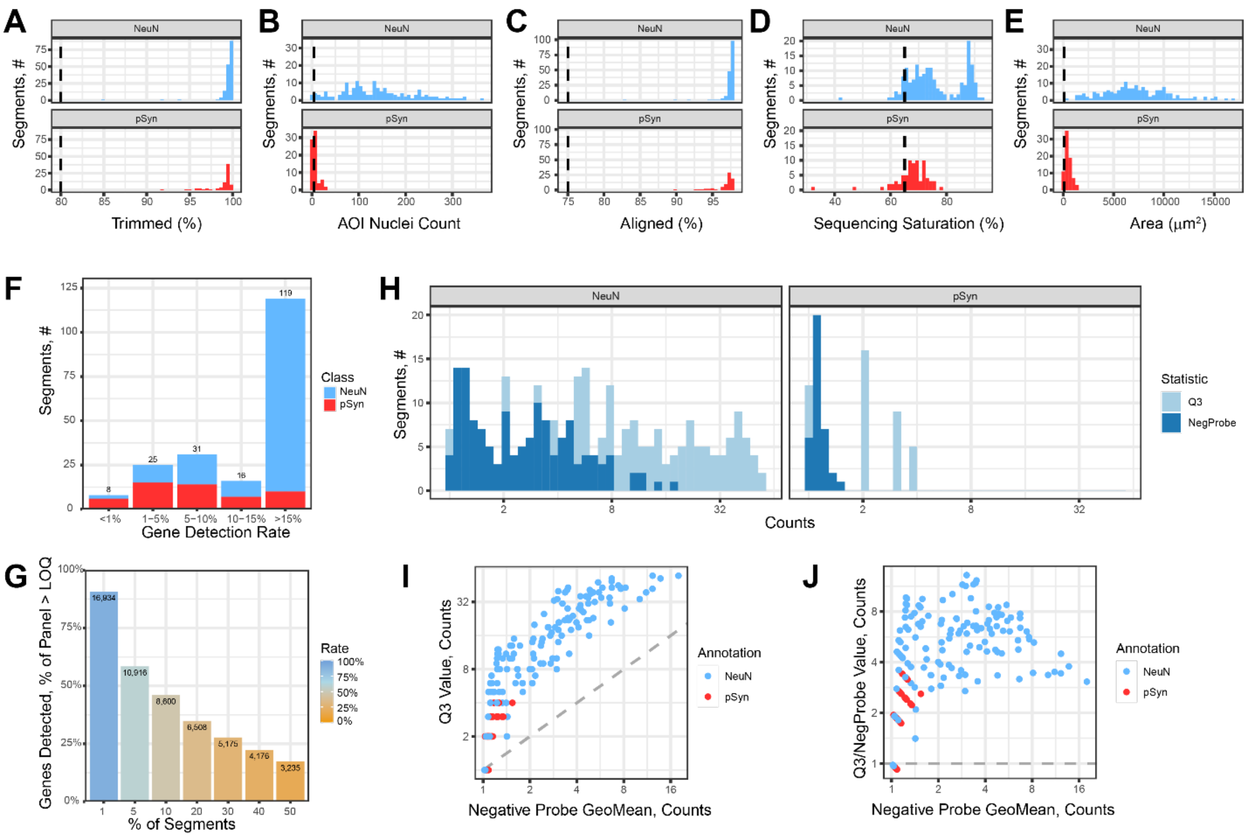

Supplementary Fig. 8 Quality control of human GeoMx data (A-E) Assessment of segments for quality based on spatial information and standard transcriptomics quality control metrics. **(F)** Number of segments with the given percentage of genes detected above the limit of quantification (LOQ). **(H)** Assessment of negative probe count values and Q3 values for each segment. **(G)** Assessment of the number of genes detected above LOQ in the given percentage of segments. **(I)** Comparison of the segments Q3 count value to the geometric mean of the negative probes. **(J)** Comparison of the segments Q3 values divided by their negative probes geometric mean to the negative probe geometric mean.

bioRxiv preprint doi: https://doi.org/10.1101/2023.05.17.541144; this version posted May 17, 2023. The copyright holder for this preprint (which was not certified by peer review) is the author/funder, who has granted bioRxiv a license to display the preprint in perpetuity. It is made available under aCC-BY 4.0 International license.

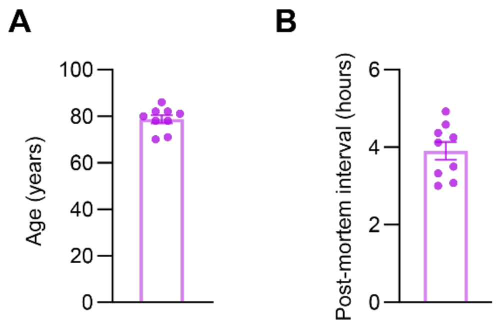

Supplementary Fig. 9 Human brain tissue information (**A**) Age and (**B**) post-mortem interval is shown for PD, PDD, and DLB cases used for GeoMx analysis.

bioRxiv preprint doi: https://doi.org/10.1101/2023.05.17.541144; this version posted May 17, 2023. The copyright holder for this preprint (which was not certified by peer review) is the author/funder, who has granted bioRxiv a license to display the preprint in perpetuity. It is made available under aCC-BY 4.0 International license.

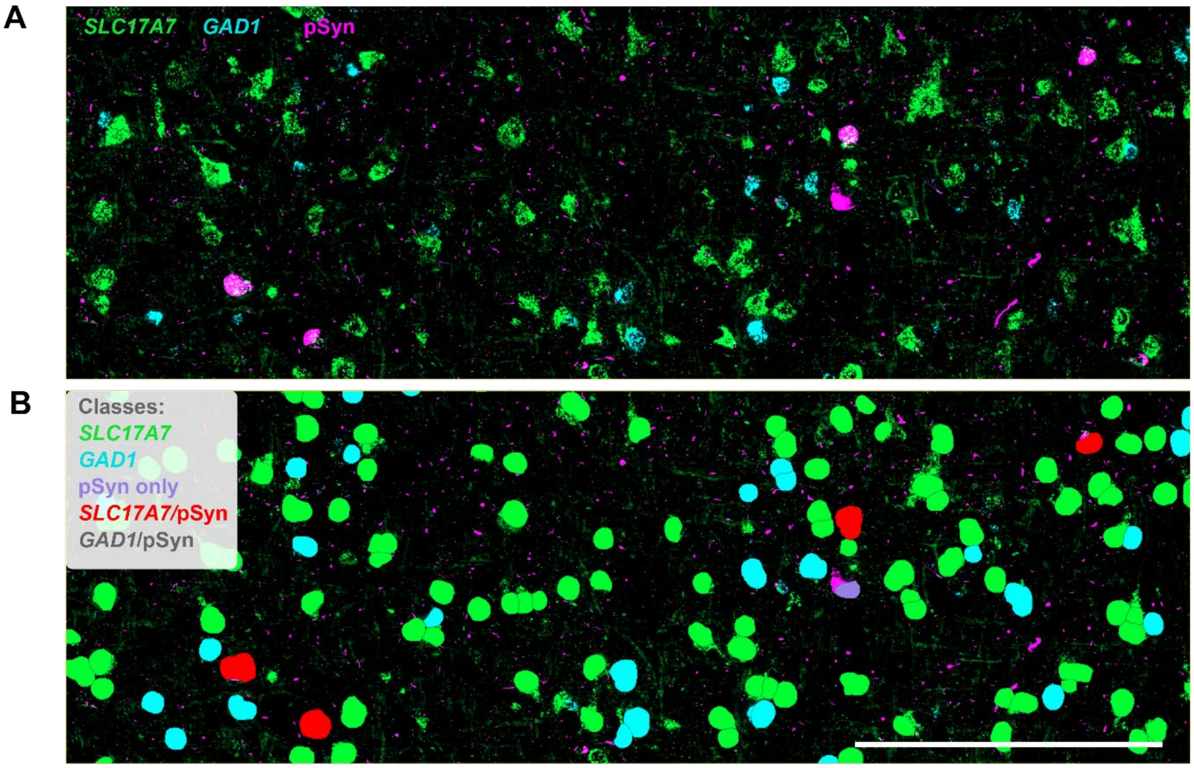

Supplementary Fig. 10 Cell classification in human cortex (**A**) Human cingulate cortex stained for RNAs *SLC17A7* and *GAD1* and protein pSyn. (**B**) The same image as in panel **A**, except cells were identified based on the presence of nuclear DAPI signal and classified based on the presence or absence of *SLC17A7*, *GAD1*, or pSyn in the cytoplasm. Cells with more than one marker were classified as having multiple markers. The overlay indicates the cell class. Scale bar = 250 µm.

bioRxiv preprint doi: https://doi.org/10.1101/2023.05.17.541144; this version posted May 17, 2023. The copyright holder for this preprint (which was not certified by peer review) is the author/funder, who has granted bioRxiv a license to display the preprint in perpetuity. It is made available under aCC-BY 4.0 International license.

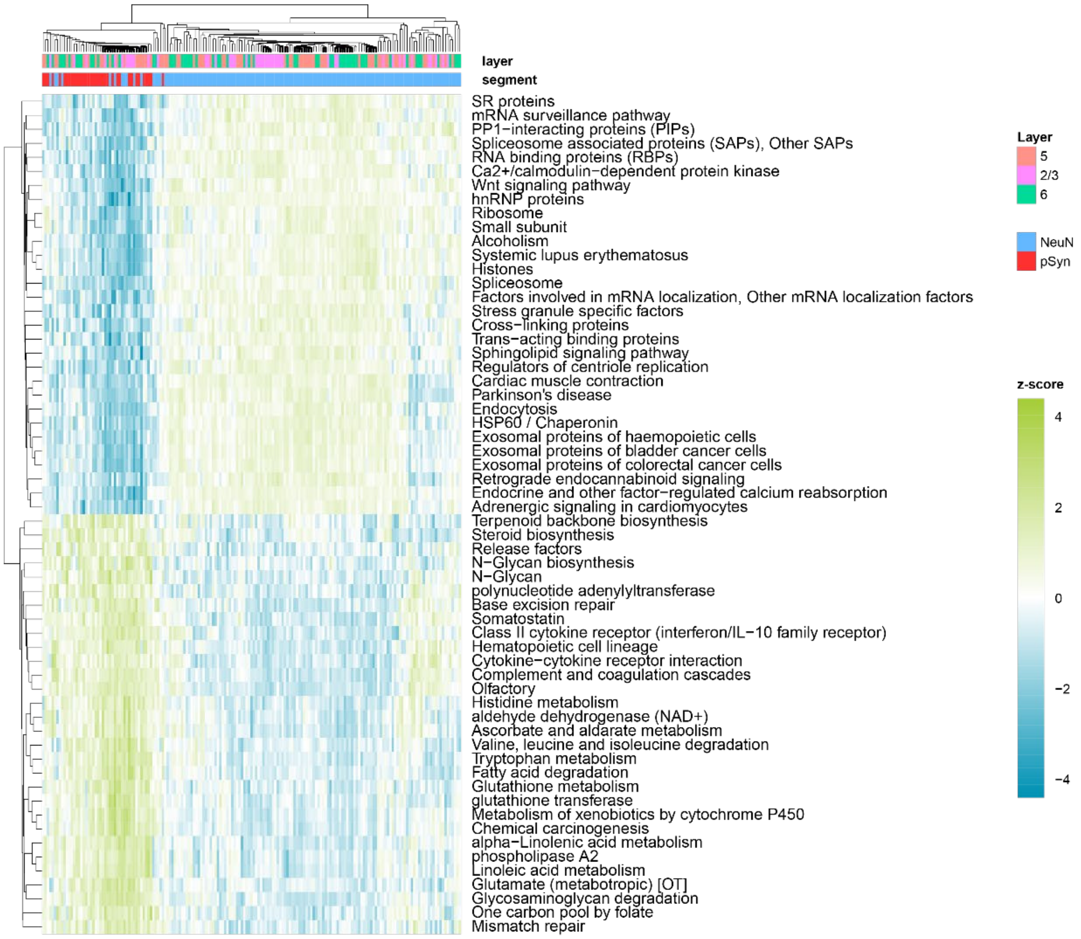

Supplementary Fig. 11 Human gene set enrichment analysis Gene set enrichment analysis was performed on NeuN and pSyn segments in from α-synuclein PFF-injected mice. Z-scores of individual segments are plotted for each pathway. The top 60 pathways enriched in either pSyn or NeuN segments are plotted.

bioRxiv preprint doi: https://doi.org/10.1101/2023.05.17.541144; this version posted May 17, 2023. The copyright holder for this preprint (which was not certified by peer review) is the author/funder, who has granted bioRxiv a license to display the preprint in perpetuity. It is made available under aCC-BY 4.0 International license.

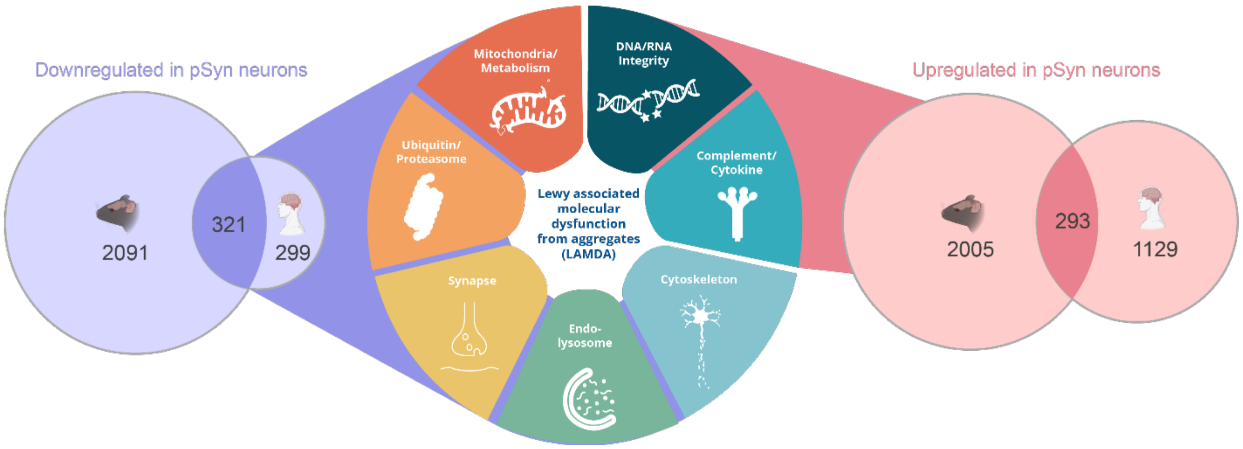

Supplementary Fig. 12. Lewy-associated molecular dysfunction from aggregates (LAMDA) Numbers of genes downregulated or upregulated in pSyn inclusion-bearing neurons are plotted from mouse and human tissue. The overlapping area is representative of the genes which show conserved expression changes in mice and humans. Conserved upregulated and downregulated genes in mouse and human fall with certain pathways and are described as a Lewy associated molecular dysfunction from aggregates (LAMDA) signature.
